# Composite Paraganglioma of the Celiac Trunk: A Case Report and a Comprehensive Review of the Literature

**DOI:** 10.3389/fsurg.2022.824076

**Published:** 2022-02-22

**Authors:** Georgios Tzikos, Alexandra Menni, Angeliki Cheva, Ioannis Pliakos, Anastasia Tsakona, Stilianos Apostolidis, Ioannis Iakovou, Antonios Michalopoulos, Theodosios Papavramidis

**Affiliations:** ^1^1st Propedeutic Department of Surgery, Aristotle University of Thessaloniki, AHEPA University Hospital, Thessaloniki, Greece; ^2^Pathology Department, Faculty of Medicine, Aristotle University of Thessaloniki, Thessaloniki, Greece; ^3^3rd Laboratory of Nuclear Medicine, Aristotle University of Thessaloniki, Thessaloniki, Greece

**Keywords:** composite paraganglioma, ganglioneuroma, celiac trunk, case, neuroendocrine tumors

## Abstract

**Introduction:**

Composite paragangliomas consist of two components, paraganglioma and ganglioneuroma, representing a rare subgroup of paragangliomas. The purpose of the study is to describe a case of composite paraganglioma of the celiac trunk and a brief review of the existing literature.

**Case Presentation:**

A 64-year-old female patient with a history of epigastric abdominal pain and a 51 mm-diameter tumor found in a Computerized Tomography of the abdomen was admitted to our surgical department for further evaluation and treatment. After a brief preoperative surgical assessment, the patient underwent a mini-laparotomy for the excision of this tumor. After having the results of the pathology report, a comprehensive review of the international literature was carried out by applying the appropriate search terms.

**Results:**

As it was found intraoperatively, the tumor was located at the cephalad aspect of the common hepatic artery, over the portal vein and the inferior vena cava. A negative-margin resection was achieved and the tumor was sent for pathology analysis. The final pathology report revealed a composite paraganglioma, with α paraganglioma and a ganglioneuroma component. Seventeen cases of extra-adrenal composite paraganglioma have been reported in the international literature so far. This case was the first one found in the area of the celiac trunk.

**Conclusions:**

Composite paragangliomas comprise rare and potentially malignant tumors with variable prognosis. Establishing their diagnosis promptly is of vital significance. Due to the first-described location of the composite paraganglioma in our case, the differential diagnosis of tumors in this area should also include composite paragangliomas.

## Introduction

Pheochromocytomas are rare chromaffin catecholamine-secreting tumors, usually located within the adrenal glands. However, when these tumors arise outside of the adrenal glands, they are defined as paragangliomas. Paragangliomas may occur anywhere along the path of the autonomic ganglia, from the base of the skull to the urinary bladder. Composite paragangliomas, a subtype of paragangliomas, usually consist of the paraganglioma and the ganglioneuroma component. Composite paragangliomas are estimated to be around 3% of the adrenal paragangliomas, being very rare especially outside the adrenal glands (1). They are reported to be found usually in the adrenal glands and less frequently in the mediastinum, in the Zuckerkandl organ at the abdominal aortic bifurcation, in the retroperitoneum, in the urinary bladder, and the central nervous system (2, 3). Herein, we present a case of composite paraganglioma located on the right of the celiac trunk, over the common hepatic artery.

## Case Presentation

**Figure 1:**
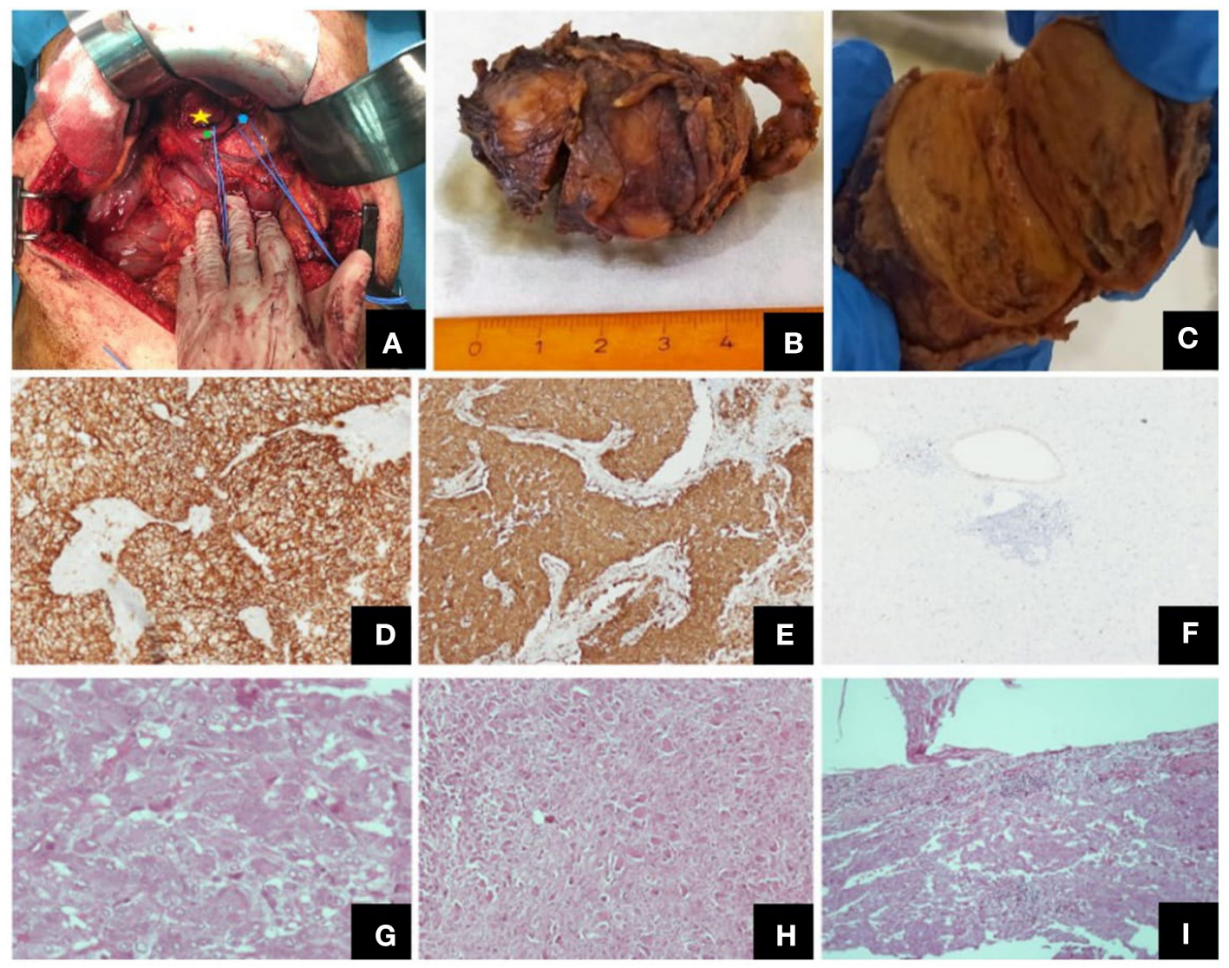
Different views of the tumor (intraoperatively, macroscopically after excision) and its histological features. **(A)** Intraoperative view of the tumor (yellow star: the tumor, green point: common hepatic artery, blue point: left gastric artery), **(B)** Macroscopic view of the excised tumor, **(C)** Tumor on vertical cross-section, where both the paraganglioma and the ganglioneuroma components were identified, Representative images of histological features: **(D)** CD56, ×100, **(E)** synapt (×100), **(F)** Ki67 labeling index (×40), **(G)** paraganglioma, H + E, (×400), **(H)** ganglioneuroma, H + E, (×100), **(I)** tumor invasion of the capsule, H + E, (×100).

A 64-year-old female patient was referred to our outpatient department for surgical evaluation due to paroxysmal epigastric abdominal pain, with mild deterioration after movement and exercise. The patient's medical history referred that she underwent total thyroidectomy, with central and left lateral compartment dissection due to thyroid papillary cancer with nodal metastasis (pT3b(m)N1Mx based on The American Joint Committee on Cancer staging) one year before her admission to our clinic. Moreover, she had already visited a Gastroenterologist, who suggested her undergoing endoscopy of the upper gastrointestinal (GI) system and a Computerized Tomography (CT) scan. The report of the upper GI endoscopy referred that mild esophagitis and gastritis were present while the CT scan revealed a 51 mm-diameter solid mass at the site of hepatogastric ligament applying pressure on the abdominal aorta. Our findings during the initial physical examination and laboratory test were normal (Table 1). Next, to identify the nature of this mass, we suggested that she should undergo an endoscopic ultrasound during which biopsies from the bulging mass would be received and sent for pathology and immunohistochemical analysis. The differential diagnosis of pathology report suggested a ganglioneuroma or a neuroendocrine neoplasm and the further immunohistochemical analysis reported that the mass included neoplasmatic cells being positive to S100 protein and also cells with positive immunostaining for neurofibers (NF), two findings which both were indicative of ganglioneuroma. Under the probable diagnosis of a ganglioneuroma, we decided to evaluate its activity regarding the secretion of catecholamines or other neurotransmitters. As a result, an analysis for creatinine, total catecholamines, metanephrines, and vanillylmandelic acid of a 24-h urine collection was performed, without revealing any abnormality (Table 1). In addition, the patient underwent screening for mutations in the ret proto-oncogene from the patient's genomic deoxyribonucleic acid (DNA) for any genetic disorders to be found, which did not detect any mutation in exons 5, 8, 10, 11, 13, 14, 15, and 16. Moreover, it was of great importance, a more comprehensive radiological assessment to be held, due to mass' special location. Thus, the patient underwent chest CT scan and abdominal CT Angiography scan with 3D reconstruction of the celiac trunk which reported again the known tumor of about 51 mm diameter, located between the aorta and the inferior vena cava in the hepatogastric space. Based on all the before mentioned data, we decided, after consensus, to suggest the patient undergoing surgical excision of the tumor due to its potential for malignancy. The patient's decision was congruent with our suggestion and she underwent a laparotomy and surgical excision of the mass. Intraoperatively, the tumor was found at the cephalad aspect of the common hepatic artery, over the portal vein, and the inferior vena cava. A negative-margin resection was achieved and the tumor was sent for pathology analysis. In addition, all the nodal tissue around the celiac trunk was excised after skeletonizing the vessels (Figure 1). The postoperative period was uncomplicated and the patient was discharged the 2nd postoperative day. The final pathology report revealed that the tumor included two masses, a sub round one enveloped by capsule and sized 6.0 × 4.2 × 3.7 cm, and a second smaller one attached to the first's outer surface. It was presented with neoplastic features and consisted of large, irregularly shaped cells. The number of nuclei was >5/10 per visual field, while in some areas they formed “zellballen balls,” and the cytoviscosity was considered as moderate to maximum. In addition, in some areas the ganglion cell population did not mix with the upper cellular findings, but appeared to be of neurogenic origin, as evidenced by immunohistochemical staining for protein S100 and CD56. Furthermore, in some areas a capsule was identified, and it was disrupted by the neoplastic cells. In these areas, neoplasmatic embolisms were identified inside the thin-walled vessels. However, the wall of the vessels was not found to be positive for immunohistochemical staining regarding CD34 and D2-40. Moreover, the neoplasmatic cells were positive for CD56, synaptophysin, and chromogranin and their nuclei presented also positive for ATRX staining, while p53 protein was identified only in <5% of them. However, the cells were negative for neurofibrins, inhibin, Melan A and HMB-45. In conclusion, the superior morphological and immunohistochemical findings were consistent with a composite paraganglioma and to a small limited extent, a ganglioneuroma. In addition, the particular histological features of the paraganglioma (diffuse growth, capsular and vascular infiltration, Ki67 cell proliferation index >2%, tumor size >5 cm) classify the neoplasm as a high metastatic potential one, according to the GAPP classification system. Two months after the procedure, the subsequent imaging evaluation of the patient, with chest and abdominal CT scan and ultrasound of the upper abdomen, did not reveal any pathological findings, except for a new-described small cyst at the head of the pancreas possibly due to chronic pancreatic inflammation. The laboratory tests were normal as well.

**Table 1:** Patient's laboratory results.

**Test**	**Value**	**Normal values**
*Complete blood count* Hematocrit	40.2%	42.0–54.0%
Hemoglobin	13.9 gr/dl	13.0–18 gr/dl
Red blood cell count	4.56 M/ml	4.5–5.5 M/ml
Mean Corpuscular Volume (MCV)	88.2 fl	78.0–98.0 fl
Mean Corpuscular Hemoglobin (MCH)	30.5 pg	27.0–31.0 pg
Mean Corpuscular Hemoglobin Concentration (MCHC)	34.6 gr/dl	32.0–36.0 gr/dl
Red Blood Cell Distribution Width (RDW-CV)	14.3%	11.5–14.0%
White Blood Cell Count	6.45 K/μL	4.0–11.0 K/μL
Neutrophils	75.7%	40.0–70.0%
Platelet Count	262 K/μL	142–450 K/μL
*Biochemical Tests* Serum glucose	96.5 mg/dl	70–105 mg/dl
Serum Urea	28.56 mg/dl	19.00–44.00 mg/dl
Serum Creatinine	0.72 mg/dl	0.72–1.25 mg/dl
Aspartate Transaminase (AST)	20.4 U/L	5.0–34.0 U/L
Alanine Transaminase (ALT)	20.7 U/L	00.0–55.0 U/L
Gamma-glutamyl Transferase	8.4 U/L	12.0–64.0 U/L
Alkaline Phosphatase (ALP)	63.4 U/L	40–150 U/L
Lactate Dehydrogenase (LDH)	190.3 U/L	125–220 U/L
C Reactive Protein (CRP)	0.039	<0.5 mg/dl
Serum Potassium	4.23 mmol/L	3.4–5.1 mmol/L
Serum Sodium	143.3 mmol/L	136.0–145.0 mmol/L
*Hormonal Assay* Triiodothyronine (T3)	1.18 ng/ml	0.6–1.6 ng/ml
Thyroxine (T4)	6.53 μg/dl	4.87–11.72 μg/dl
Thyroid Stimulating Hormone (TSH)	1.43 μIU/ml	0.35–4.94 μIU/ml
Serum Free Metanephrine	0.31	<0.50 nmol/L
Serum Free Normetanephrine	0.56	<0.90 nmol/L
*Serology* Surface antigen of the hepatitis B virus (HBsAg)	0.11 S/CO	Negative < 1 .00 S/CO
Hepatitis C Virus test	0.09 S/CO	Negative < 1.00 S/CO
HIV Ag/Ab	0.11 S/CO	Negative < 1.00 S/CO
*Coagulation Tests* activated Partial Thromboplastin Time (aPTT)	31.6 sec	25.0–45.0 sec
Prothrombin Time (PT)	11.55 sec	12.00–14.00 sec
International Normalized Ratio (I.N.R.)	0.87	1.00–1.50
*24-h urine collection* Creatinine	768 mg/24 h	600–1,800 mg/24 h
Total Catecholamines	213 μg/24 h	65–515 μg/24 h
Adrenaline	11.3 μg/24 h	0.0–20.0 μg/24 h
Noradrenaline	62.7 μg/24 h	15.0–80.0 μg/24 h
Total Metanephrines	0.87 μg/ mg of creatinine	<1.20 μg / mg of creatinine
Vanillylmandelic Acid	3.4 mg/24 h	<11.0 mg / 24 h

## Discussion

Pheochromocytomas are tumors which in their majority originate from the chromaffin cells of the adrenal medulla (85–90%) and less frequently from the extra-adrenal sympathetic nerve tissue, mainly paraspinal or paraaortic, called paragangliomas (4). They can be either sporadic or familial (5). The usual location for paragangliomas is primarily in the head, the cervix, or the mediastinum (6), usually secreting catecholamines. Similar to common paragangliomas, the composite paragangliomas are primarily functional, secreting catecholamines such as adrenaline, noradrenaline, and dopamine or corticotrophin-releasing hormone (CRH) as well (7). Based on this fact, common symptoms are severe headache, nausea, palpitations, sweating, fatigue, permanent or paroxysmal hypertension, or even orthostatic hypotension. Moreover, pallor, redness of the face, weight loss, and hyperglycemia are common signs as well (8). However, in our case, since the composite paraganglioma was not an active one, the patient did not experience any of the relating with catecholamines-secretion symptoms, but only a feeling of abdominal tenderness and pain especially right after exercise.

Ten percent of all the pheochromocytomas have a chance of malignancy, with extra-adrenal gland localization advocating a particularly high rate of malignancy and metastatic disease (9). On the other hand, ganglioneuromas are considered to be the most common form of neuroblastoma, mainly in young adults (10). They are primarily retroperitoneal and tend to be asymptomatic until they become large enough to give symptoms by pressing nearby structures (11). However, some ganglioneuromas may be also functional and secrete peptides, such as VIP and somatostatin, causing diarrhea, hypertension, and sweating.

Regarding composite paragangliomas, they are considered to be rare tumors. In about 70% of them, paraganglioma coexists with a ganglioneuroma component. They affect patients around 40 to 60 years old, with equal distribution across males and females (12). The size of composite paragangliomas ranges from 1 to 35 cm (13). In our case, the size of the tumor was considered to be an average one, about 6 × 4.2 × 3.7 cm (the volume was about 80 cc).

Fewer than 70 cases have been reported in the medical literature, most of which are located in the adrenal glands, while the extra-adrenal composite tumors have been reported only occasionally (14). In particular, only 17 extra-adrenal cases have been described in the literature so far (Table 2). From them 5 were found in the urinary bladder, 6 in the retroperitoneum, 1 in the neck, 1 in the duodenum, 1 in the pancreas, 1 in the filum terminale, 1 in the caude equine and 1 case of spinal and pelvic bone metastatic lesions. The mean age of all these cases was 58.8 years old, whereas 10 of them were females and 6 were males (In one case the gender was not reported). Thus, to our knowledge, in this study we describe the first case demonstrating a composite paraganglioma-ganglioneuroma located in the area near the celiac trunk, cephalad to the common hepatic artery.

**Table 2:** Extra-adrenal composite paragangliomas referred in the literature.

**Case**	**Title**	**First author**	**Year of publication**	**Location of the composite paraganglioma**	**Age (years)**	**Gender**	**Size (mm)**	**Symptoms**	**Activity (preoperatively)**
1	Composite Paraganglioma-Ganglioneuroma of the Urinary Bladder: A Clinicopathologic, Immunohistochemical, and Ultrastructural Study of a Case and Review of the Literature	King-Yin Lam	1998	Urinary bladder	81	Female	NR^*^	Whole-stream painless hematuria	NR^*^
2	Pigmented composite paraganglioma-ganglioneuroma of the urinary bladder	Pavel Dundr	2003	Urinary bladder	70	Female	65(diameter)	NR^*^	NR^*^
3	Composite paraganglioma-ganglioneuroma of the urinary bladder	Hiroyuki Usuda	2005	Urinary bladder	73	Male	40 × 30 × 25	Dysuria	Elevated serum catecholamine and elevated VMA^**^ and catecholamine in 24-hour urine collection
4	Composite paraganglioma-ganglioneuroma of the urinary bladder: a rare neoplasm causing hemodynamic crisis at tumor resection	C-H Chen	2009	Urinary bladder	64	Male	50 × 40 × 30 (Residual tumor: 18 × 11 × 30)	Gross painless hematuria	Not measured
5	Composite Paraganglioma and Neuroblastoma of the Urinary Bladder: A Rare Histopathological Entity	Evan Lacefield	2015	Urinary bladder	45	Male	44(diameter)	Flank, abdominal pain and dysuria	Elevated serum normetanephrine, urine VMA^**^, urine norepinephrine and chromogranin A
6	Composite Paraganglioma: Pioneering in the Head and Neck	Santiago Delgado	2019	Neck	50	Female	59 × 12 × 5	Incidental finding of an enhancing mass in the right carotid space, (8 months after the first diagnosis) neck pain, anxiety, and episodes of dizziness,	24-hour urine catecholamine levels, including epinephrine, norepinephrine and dopamine were measured to be within normal range
7	Composite paraganglioma-ganglioneuroma in the retroperitoneum	Shoji Hirasaki	2009	Retroperitoneum	63	Female	65 × 50 × 30	Left femoral shaft fracture and left leg edema	Serum adrenaline, noradrenaline and dopamine were measured to be within normal range
8	Composite paraganglioma with ganglioneuroma in the retroperitoneal space	Hideaki Ito	2010	Retroperitoneum	31	Female	60 × 50 × 46	Referral for evaluation of pulmonary embolism after she had a scheduled Cesarean section at 37 weeks of pregnancy. Incidental finding of the mass after CT	NR^*^
9	Adrenal and Extra-Adrenal Non-functioning Composite Pheochromocytoma/Paraganglioma with Immunohistochemical Ectopic Hormone Expression: Comparison of Two Cases	Jing Gong	2010	Retroperitoneum	50	Male	45 × 40 × 25	Dull back pain for 3 months	Serum catecholamine levels and consecutive 2-day measurements of 24-hour urine catecholamine levels were measured to be within normal range
10	Composite paraganglioma and ganglioneuroma in the retroperitoneum: a case report	Yuji Ohtsuki	2012	Retroperitoneum	68	Female	30 × 22 × 20	Abdominal pain for 4 months	NR^*^
11	Retroperitoneal composite pheochromocytoma-ganglioneuroma: a case report and review of literature.	Jinchen Hu	2013	Retroperitoneum	52	Female	60 × 50 × 40	Watery diarrhea and febricity for one day, palpitation and debilitation for 6 hours	Not measured
12	Composite paraganglioma-ganglioneuroma concomitant with adrenal metastasis of medullary thyroid carcinoma in a patient with multiple endocrine neoplasia type 2B: A case report	Mutsushi Yamasaki	2016	Retroperitoneum	59	Male	30(diameter)	Multiple endocrine neoplasia type 2B (MEN2B)	Elevated 24-hour urinary metanephrine and VMA^**^
13	Extra-adrenal Composite Paraganglioma with Ganglioneuroma Component Presenting as a Pancreatic Mass	Frediano Inzani	2009	Pancreas	57	Female	30 × 30 × 25	Hypertension	Not measured
14	Paraganglioneuroma of the duodenum: an evolutionary hybrid?	T Cooney	1977	Duodenum	65	Female	10(diameter)	Incidental finding of the mass during necropsy	Not measured
15	Ganglioneuromatous paraganglioma of the cauda equina—a pathological case study	Peter Pytel	2005	Cauda equina	74	Female	18(diameter)	Back and leg pain without any weakness or other neurological deficits	NR^*^
16	Composite ganglioneuroma-paraganglioma of the filum terminale	Ganesh M. Shankar	2010	Filum terminale	47	Male	26 × 17 × 12	8-week history of worsening lower-back pain, intermittent tingling sensation in the inguinal area and painful bowel movements. Hypertension.	NR^*^
17	Recurrent multiple spinal paragangliomas as a manifestation of a metastatic composite paraganglioma-ganglioneuroblastoma	Jens Gempt	2013	Spinal and pelvic bone metastatic lesions	51	NR^*^	Not resected, only biopsies obtained from the bone marrow	Low back pain, radicular and progressive ataxia	NR^*^

* *NR, Not Reported*.

** *VMA, Vanillylmandelic Acid*.

Composite paragangliomas are often associated with familial neoplasm syndromes, such as neurofibromatosis type 1 (NF1) or multiple endocrine neoplasia type 2 (MEN2) (15). In multiple endocrine neoplasia type 2 (MEN2), an autosomal-dominant cancer syndrome with major components of medullary thyroid carcinoma (MTC), pheochromocytoma, and hyperparathyroidism, about 50% of the patients develop pheochromocytomas, located, almost always, in the adrenal medulla. Moreover, extra-adrenal pheochromocytomas (paragangliomas) are unusual (about 3% of the cases), while the co-existence of a composite paraganglioma-ganglioneuroma with any kind of MEN2-familial syndromes is quite rare (5). In our case, any association of this particular composite tumor was not confirmed, nor any kind of familial syndrome, based on the results of the genetic screening. However, it is worth mentioning that the patient has already undergone total thyroidectomy due to papillary, not medullary, metastatic cancer. In addition, in the postoperative imaging follow-up, a mass in the area of the head of the pancreas was found, constituting a point of concern, although it is currently attributed to a benign cystic lesion.

Imaging studies for the diagnosis of paragangliomas include CT or Magnetic Resonance Imaging (MRI) scan, while scintigraphy may be an alternative utility when these imaging tests fail to localize the tumor. Abdominal CT scan has an accuracy of about 85–95% for detecting tumors with a threshold size of 1 cm (16). Most of the pheochromocytomas reveal CT attenuation of more than 10 Hounsfield Units (HU) but sometimes it is quite difficult to differentiate a pheochromocytoma from another adenoma or adrenal metastasis (17). On the other hand, MRI is reported to have a sensitivity of about 100% in detecting adrenal pheochromocytomas, and in about 70% of T2-weighted images, the tumors appear hyperintense due to their water content or internal hemorrhage (18). Furthermore, scanning with ^123^iodine-labeled metaiodobenzylguanidine (MIBG) may be helpful for cases in which CT or MRI are inconclusive, even though the pheochromocytoma has already been proven biochemically. Its specificity is reported to be between 82 and 92% and its sensitivity ranges widely, from 53 up to 94% (19).

In this case, we would like to emphasize the difficulty of establishing the diagnosis of paraganglioma preoperatively. The laboratory tests were indicative and the ultrasound imaging revealed a mass, without any special characteristic indicative of its origin. Moreover, an ultrasound-guided biopsy of this tumor suggested the diagnosis of a ganglioneuroma and not a paraganglioma. In addition, images obtained by CT scan were not characteristic of a paraganglioma, while CT-angiography just helped us identify the relations of the tumor with the celiac artery and the other structure of this area. As a result, the definite diagnosis of the composite paraganglioma was established only after the final pathology analysis of the excised tissue, and besides, it is very common to have the precise diagnosis for this kind of tumors only after the pathology examination have been completed (20).

The prognosis of composite paragangliomas varies and depends on the existence of malignancy. For non-malignant disease, the 5-year survival rate is more than 95%. However, in patients with malignancy, the 5-year survival rate is <50% (21). Dhir et al. reported that the likelihood of malignant disease is greater among younger patients, having a larger-sized tumor or being diagnosed with paraganglioma, as well as in patients with mutations in succinate dehydrogenase complex (SDHD) gene (22). Metastatic lesions are almost always derived from the neural component. Pheochromocytoma metastases, as a single entity or in conjunction with the malignant neural component, were found in some uncommon cases in which liver metastatic lesions were reported deriving from a composite paraganglioma (23). Fortunately, in our case, a locally advanced tumor according to imaging examination was not confirmed. However, based on the histopathological and immunohistochemical characteristics, this composite paraganglioma of our patient is considered to have high malignancy potential, based on the GAPP score classification (Table 3). According to the detailed pathology report, the excised tumor had some foci of “zellballen and pseudorosette-forming” pattern, moderate to high cellularity, vascular and capsular invasion, Ki-67 immunoreactivity more than 1%, and also coagulation necrosis. In addition, the immunohistochemical analysis reported that the tumor was found to be positive in protein S100, which is also associated with a worse prognosis. As a result, this tumor scored 6–7 points and was classified as a tumor with moderate to low differentiation and of high metastatic risk (24). This is the reason why a very strict active surveillance of the patients is mandatory. Three months postoperatively, imaging examinations with CT scan and ultrasound were completed and no recurrent or metastatic disease has been documented so far.

**Table 3:** The grading system for adrenal pheochromocytoma and paraganglioma (GAPP score).

**GAPP parameters**	**Points scored**
*Histological pattern*	
Zellballen	0
Large and irregular cell nest	1
Pseudorosette (even focal)	1
*Comedo-type necrosis*	
Absence	0
Presence	2
*Cellularity*	
Low (<150 cells/U)	0
Moderate (150–250 cells/U)	1
High (>250 cells/U)	2
*Ki67 labeling index (%)*	
<1	0
1–3	1
>3	3
*Vascular or capsular invasion*	
Absence	0
Presence	1
*Catecholamine type*	
Non-functioning	0
Adrenergic type	0
Noradrenergic	1
**Total maximum score**	**10**

In conclusion, composite paragangliomas comprise rare and potentially malignant tumors with variable prognosis. Establishing their diagnosis promptly is of vital significance. Based on our case, due to the first-described location of a composite paraganglioma near the celiac artery, the differential diagnosis of the tumors found in this area should include composite paragangliomas as well.

## Author Contributions

GT and AMe were the chief investigators, wrote the manuscript, and collected the majority of the data. AC and AT conducted the histopathological and immunohistochemical analysis. IP and SA were the surgeons who conducted the excision of the tumor. II collected some additional data for the study. TP wrote and corrected the manuscript for its scientific basis. AMi was the director of the Department of Surgery and provided his permission for this study. All authors have read and approved the final manuscript.

## Conflict of Interest

The authors declare that the research was conducted in the absence of any commercial or financial relationships that could be construed as a potential conflict of interest. The reviewer SM declared a shared affiliation with all the authors to the handling editor at the time of the review.

## Publisher's Note

All claims expressed in this article are solely those of the authors and do not necessarily represent those of their affiliated organizations, or those of the publisher, the editors and the reviewers. Any product that may be evaluated in this article, or claim that may be made by its manufacturer, is not guaranteed or endorsed by the publisher.
